# Whole-transcriptome analysis of flow-sorted cervical cancer samples reveals that B cell expressed TCL1A is correlated with improved survival

**DOI:** 10.18632/oncotarget.4526

**Published:** 2015-06-19

**Authors:** Simone Punt, Willem E. Corver, Sander A.J. van der Zeeuw, Szymon M. Kielbasa, Elisabeth M. Osse, Henk P.J. Buermans, Cornelis D. de Kroon, Ekaterina S. Jordanova, Arko Gorter

**Affiliations:** ^1^ Department of Pathology, Leiden University Medical Center, Albinusdreef, Leiden, The Netherlands; ^2^ Department of Sequencing Analysis Support Core, Leiden University Medical Center, Albinusdreef, Leiden, The Netherlands; ^3^ Department of Bioinformatics Center of Expertise, Leiden University Medical Center, Albinusdreef, Leiden, The Netherlands; ^4^ Department of Leiden Genome Technology Center, Leiden University Medical Center, Albinusdreef, Leiden, The Netherlands; ^5^ Department of Gynaecology, Leiden University Medical Center, Albinusdreef, Leiden, The Netherlands; ^6^ Center for Gynecological Oncology Amsterdam, VUMC, De Boelelaan, Amsterdam, The Netherlands

**Keywords:** uterine cervical cancer, tumor microenvironment, immune response, B lymphocytes, TCL1A

## Abstract

Cervical cancer is typically well infiltrated by immune cells. Because of the intricate relationship between cancer cells and immune cells, we aimed to identify both cancer cell and immune cell expressed biomarkers. Using a novel approach, we isolated RNA from flow-sorted viable EpCAM^+^ tumor epithelial cells and CD45^+^ tumor-infiltrating immune cells obtained from squamous cell cervical cancer samples (*n* = 24). Total RNA was sequenced and differential gene expression analysis of the CD45^+^ immune cell fractions identified *TCL1A* as a novel marker for predicting improved survival (*p* = 0.007). This finding was validated using qRT-PCR (*p* = 0.005) and partially validated using immunohistochemistry (*p* = 0.083). Importantly, TCL1A was found to be expressed in a subpopulation of B cells (CD3^−^/CD19^+^/CD10^+^/CD34^−^) using multicolor immunofluorescence. A high *TCL1A/CD20* (B cell) ratio, determined in total tumor samples from a separate patient cohort using qRT-PCR (*n* = 52), was also correlated with improved survival (*p* = 0.027). This is the first study demonstrating the prognostic value of separating tumor epithelial cells from tumor-infiltrating immune cells and determining their RNA expression profile for identifying putative cancer biomarkers. Our results suggest that intratumoral TCL1A^+^ B cells are important for controlling cervical cancer development.

## INTRODUCTION

Cervical cancer is caused by persistent infection with human papillomavirus (HPV) and is currently the fourth-leading cause of cancer-related death worldwide [1]. The prognosis for patients with cervical cancer relapse is poor. Although the host's immune system can usually clear an HPV infection, chronic inflammation can contribute to tumorigenesis [2]. Cervical cancer is characterized by a large number of infiltrating immune cells, and observational and functional studies have revealed that several immune cell types can play a crucial role in the prognosis of patients with cervical cancer. A high number of cytotoxic T cells and T helper type 1 cells is correlated with improved survival in several cancer types [3, 4], including cervical cancer [5]. Cytokines derived from immune cells determine the type of immune response, but they can also directly promote tumor growth [6]. For example, our group and others have shown that a high level of IL-6 expression is an independent predictor of poor survival [7–9]. The type of immune infiltrate may even better predict patient prognosis than histopathological methods [10]. However, because tumor cells can also produce cytokines and suppress a tumor-targeting immune response [11], the complex interaction between tumor cells and the local microenvironment makes it challenging to discriminate between effects arising from the tumor cells and effects arising from immune cells.

Because of the intricate relationship between cervical cancer cells and the infiltrating immune cells, the infiltrating immune cells represent an opportunity to identify novel cell-specific biomarkers for improved patient survival. Immunohistochemistry (IHC) is a commonly used method for investigating separate cell types, but is suitable for studying only a select number of strongly expressed antigens. To detect global differences in gene expression, total RNA is usually studied. However, studying gene expression in tumor samples usually fails to provide information regarding the origin of the cells expressing a particular RNA. Therefore, to distinguish between genes expressed by tumor epithelial cells and genes expressed by tumor-infiltrating immune cells, these two cell subpopulations must first be separated. Although fluorescence-activated cell sorting is a commonly used strategy for selectively identifying cancer stem cells [12–14] and for studying the molecular genetics of tumor cell subpopulations [15, 16], to the best of our knowledge flow cytometry has not been used for transcriptome analysis of matched tumor cell and immune cell subpopulations.

The aim of this study was to discover potential biomarkers expressed by tumor cells or infiltrating immune cells using an unbiased approach. Tumor epithelial cells were separated from immune cells using flow cytometry. Subsequently, we performed total RNA sequencing (RNA-seq) and differential gene expression analysis to examine which genes may play a role in clinical outcome. Our analysis revealed that T-cell leukemia/lymphoma 1A (*TCL1A*) is a novel immune cell expressed marker for predicting improved survival in patients with cervical cancer. This finding was confirmed using both qRT-PCR and IHC analyses. In addition, we used multicolor immunofluorescence to demonstrate that TCL1A is expressed by a subpopulation of B cells in cervical cancer. This novel approach highlights the potential relevance of tumor infiltrating B cells.

## RESULTS

### Cervical cancer samples

The patient and tumor characteristics are summarized in Table 1. First, we determined whether our patient cohort (*n* = 24) was representative of the total squamous cervical cancer patient cohort followed from 1985 through 2005 (*n* = 173). The only significant difference identified was increased tumor size in our patient cohort (*p* = 0.008), which was due to the requirement for larger tumors to prepare cell suspensions. We found no difference between the study cohort and the other patient samples with respect to survival or postoperative radiotherapy treatment (data not shown).

**Table 1:** Patient and tumor characteristics

Clinico-pathological parameter	Category	N (%)
Age	Median	43
	Range	26-63
FIGO stage^a^	IB	20 (83)
	IIA	4 (17)
TNM stage^b^	IB1	3 (13)
	IB	1 (4)
	IB2	13 (54)
	IIA	5 (21)
	IIB	1 (4)
	IIIB	1 (4)
Lymph nodes	Negative	15 (63)
	Positive	9 (38)
Tumor size (mm)^c^	<40	4 (17)
	≥40	19 (79)
Vaso-invasion	Absent	11 (46)
	Present	13 (54)
Infiltration depth (mm)^c^	<15	8 (33)
	≥15	15 (63)
HPV type	16	13 (54)
	18	5 (21)
	Other	6 (25)

a FIGO, International Federation of Gynaecologists and Obstetricians

b TNM, classification of malignant tumors based on the tumor size and the involvement of regional lymph nodes and distant metastases

c Data were not available for all patients.

### Separation of tumor cell and immune cell fractions

**Figure 1:**
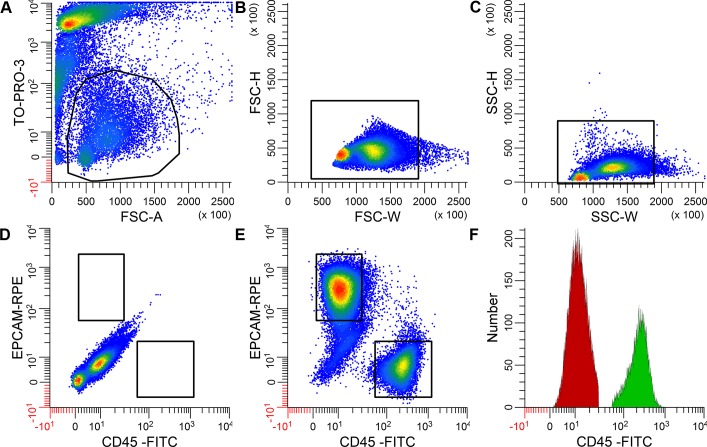
Example of EpCAM^+^ and CD45^+a^ single-positive cells obtained from a cervical cancer cell suspension using flow cytometry The negative control stained for TO-PRO-3 but not for EpCAM or CD45 is shown in **A.**-**D.** Living cells were selected based on the absence of TO-PRO-3 staining **A.**, after which cell aggregates were excluded using forward scatter width **B.** and side scatter height and width **C.** Cell sorting was based on the expression of either EpCAM or CD45 in the double stained samples **E.**, but not in the unstained control **D.** Restricted gates were used as indicated to obtain pure cell populations. Panel **F.** shows the distribution of CD45 expression in the CD45^+^ immune cells (in green) and the EpCAM^+^ tumor cells (in red). Similar results were obtained with respect to EpCAM expression (data not shown).

**Figure 2:**
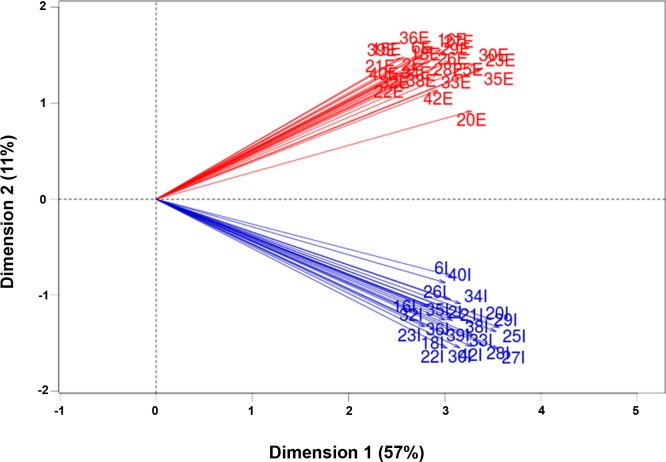
Principal component analysis of all fractions Differential gene expression of the tumor cell fractions and immune cell fractions (*n* = 46) obtained from 23 patients (one sample was excluded based on the external RNA controls) was compared using principal component analysis. The numbers indicate the individual patient-matched fractions; “E” indicates a tumor epithelial cell fraction, and “I” indicates an infiltrating immune cell fraction. The percentages in each dimension indicate the amount of variation explained by each dimension. Note that all tumor cell fractions clustered, and all immune cell fractions clustered.

Following the dissociation of fresh tumor samples, TO-PRO-3^−^EpCAM^+^CD45^−^ viable tumor epithelial cells (hereafter referred to as the tumor cell fraction) and TO-PRO-3^−^EpCAM^−^CD45^+^ viable immune cells (hereafter referred to as the immune cell fraction) were sorted using flow cytometry (Figure 1). RNA-seq data were obtained from total RNA isolated from both cell fractions. The gene expression pattern in the tumor cell fractions differed considerably from the expression pattern in the immune cell fractions, as shown by two separate clusters of all tumor cell and immune cell fractions upon performing a principal component analysis of all fractions (Figure 2).

**Figure 3:**
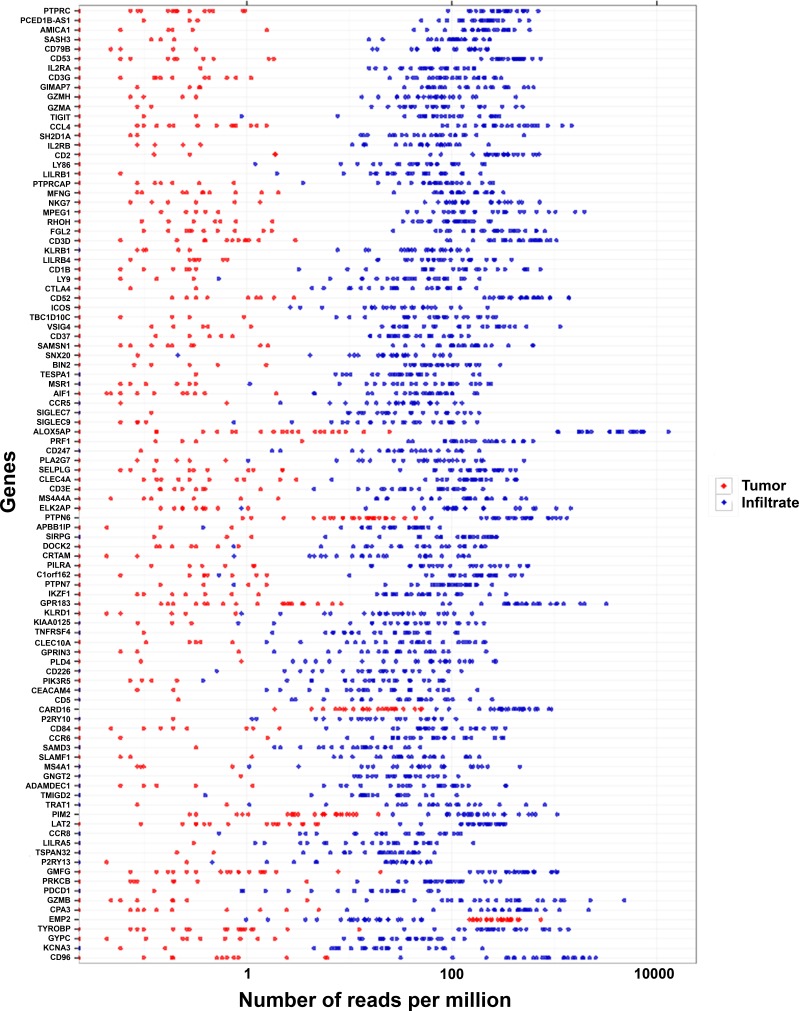
The most differentially expressed genes in the tumor versus the infiltrate fractions The 100 most significantly differentially expressed genes between the tumor cell (red dots) and infiltrate (blue dots) fractions are shown. The gene with the lowest p value (*PTPRC*) is listed at the top.

Based on p value ranking, the most upregulated gene in the immune cell fractions was protein tyrosine phosphatase receptor-type C (*PTPRC*, *p* = 6.54E-146; Figure 3, Supplemental Table S1), which is also known as *CD45*, the marker used to flow-sort the immune cells. The most significantly enriched biological process Gene Ontology (GO) terms in the immune cell fractions were immune response, leukocyte and lymphocyte terms.

In the tumor cell fractions, the most significantly enriched biological process GO terms were cell cycle, tissue development and cellular process, confirming the identity of this fraction as well. The most upregulated gene in the tumor cell fractions (i.e., with the lowest p value) was epithelial membrane protein 2 (*EMP2*, *p* = 5.49E-36; Figure 3). In addition, another highly significantly upregulated gene was *CDKN2A* (also known as *p16*; *p* = 7.08E-25), which is upregulated specifically in cervical cancer cells.

The most strongly differentially expressed genes were upregulated in the immune cell fractions compared with the tumor cell fractions, as shown by the ranking of the 100 most differentially expressed genes (Figure 3).

### Prognostic factors identified

**Figure 4:**
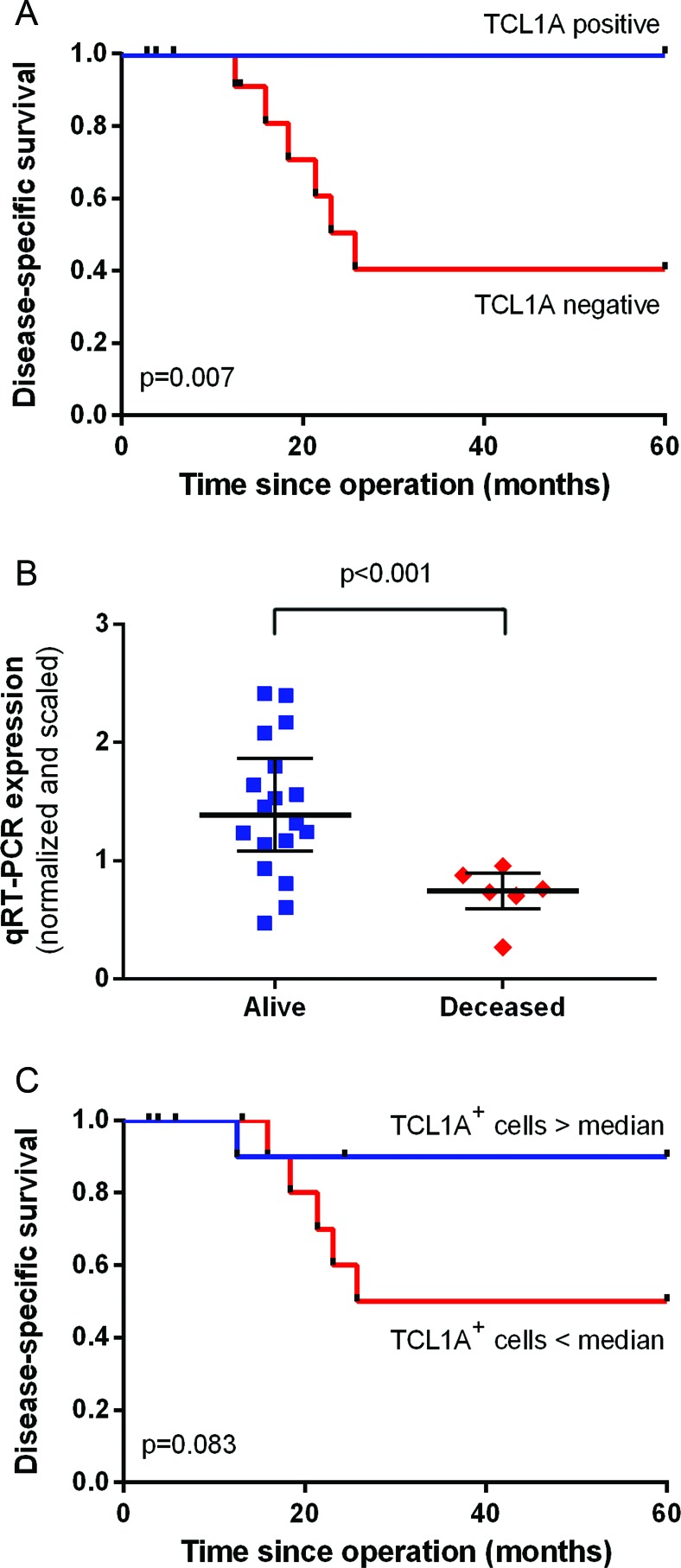
Correlation between TCL1A expression and survival **A.** Kaplan-Meier survival curve and log-rank disease-specific survival curve based on the presence or absence of *TCL1A* sequence reads. **B.** The *TCL1A* qRT-PCR expression values were compared between patients who survived and patients who were deceased five years after surgery. The median and interquartile range are depicted. **C.** Kaplan-Meier disease-specific survival analysis for a high number of TCL1A^+^ cells versus a low number of TCL1A^+^ cells based on immunohistochemistry.

Within the tumor cell fractions, no genes were significantly differentially expressed based on patient survival status five years after surgery. Among the immune cell fractions, 17 genes were significantly differentially expressed based on patient survival status (Supplemental Table S2 and Supplemental Figure S1). The most prominent gene was *TCL1A*, which encodes a lymphocyte-specific protein. Specifically, *TCL1A* was expressed in the majority of surviving patients, but it was not expressed in any of the patients who died within five years of surgery. The expression of *TCL1A* was significantly correlated with improved disease-free survival (*p* = 0.047) and disease-specific survival (*p* = 0.007; Figure 4A).

### Validation of the correlation between TCL1A expression and improved survival

To validate the technique used, we performed a qRT-PCR analysis of *TCL1A* expression using the same 24 immune cell fraction RNA samples that were used for generating the RNA-seq data. This analysis confirmed that *TCL1A* was expressed at significantly increased levels in patients who survived compared with patients who died within five years (*p* = 0.0003; Figure 4B). Our qRT-PCR analysis confirmed that *TCL1A* expression was significantly correlated with improved disease-free survival (*p* = 0.033) and improved disease-specific survival (*p* = 0.005), with Kaplan-Meier survival curves that were qualitatively similar to Figure 4A (data not shown).

To validate this correlation at protein expression level, the corresponding formalin-fixed, paraffin-embedded (FFPE) samples were stained for TCL1A using IHC. A survival analysis was performed by comparing patients with a high (i.e., above the median of 56 cells/mm^2^) versus a low (i.e., below the median) number of strong TCL1A^+^ cells (Figure 4C). One patient died despite having a high number of TCL1A^+^ cells; nevertheless, the data revealed a trend towards improved disease-specific survival (*p* = 0.083).

### Phenotypic characterization of TCL1A^+^ cells

**Figure 5:**
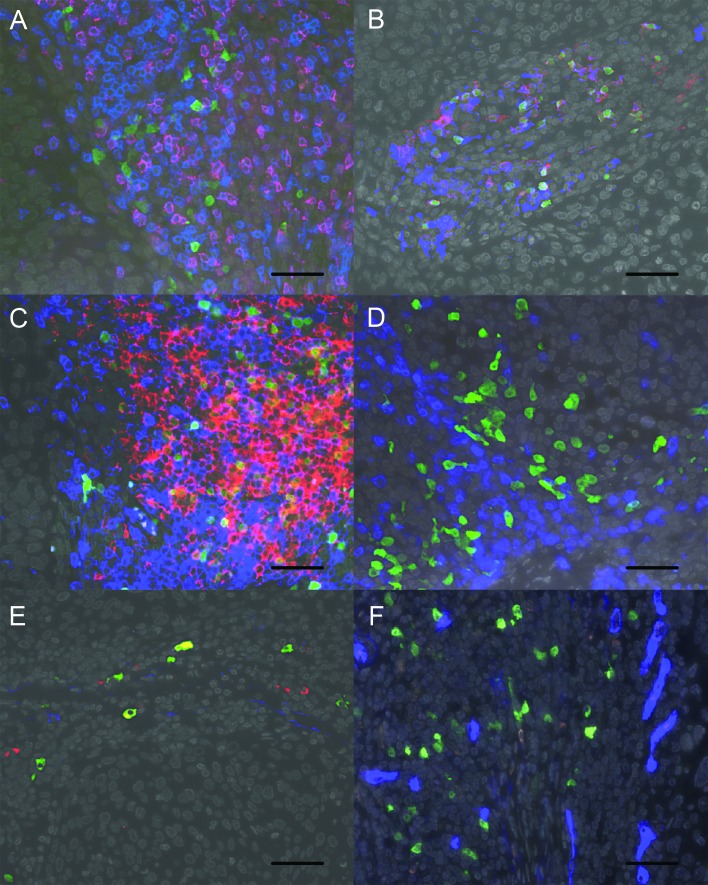
Phenotype of the TCL1A^+^ cells **A.** Representative image of a squamous cell cervical cancer FFPE specimen stained for TCL1A (green), CD8 (red) and CD3 (blue). Note that all TCL1A^+^ cells were negative for T cell markers. **B.** Staining for TCL1A (green), CD20 (red) and CD19 (blue) shows that TCL1A^+^ cells predominantly expressed CD19, and some of these cells also expressed CD20. **C.** Staining for TCL1A (green), CD20 (red) and CD10 (blue) shows that the majority of TCL1A^+^ cells expressed CD10, and some TCL1A^+^ cells expressed CD20. **D.** Relatively few TCL1A^+^ cells (green) expressed CD79a (blue). **E.** Relatively few TCL1A^+^ cells expressed DNTT (red); CD10 is shown in blue. **F.** TCL1A^+^ cells did not express CD34 (blue). In all panels, the nuclei were counterstained with DAPI (shown in grey), and the scale bar represents 50 μm.

Immunofluorescence staining was used to determine the phenotype of the TCL1A^+^ cells in FFPE samples from four patients included in the RNA-seq analysis. Strikingly, the TCL1A^+^ cells did not express CD3 or CD8 (Figure 5A), but the majority of TCL1A^+^ cells expressed the pan B cell marker CD19 (Figure 5B). Upon further investigation of the B cell phenotype, we found that the majority of TCL1A^+^ cells also expressed CD10. A smaller population of cells expressed CD20 (Figure 5B, 5C); relatively few cells expressed CD79a (Figure 5D) or DNA nucleotidylexotransferase (DNTT; Figure 5E), and none of the cells expressed the pro B cell marker CD34 (Figure 5F).

To quantify the relative frequencies of the two predominant TCL1A^+^ B cell subpopulations (i.e., TCL1A^+^CD19^+^CD20^−^ and TCL1A^+^CD19^+^CD20^+^ cells), the numbers of these cell subpopulations were scored in the FFPE samples of all 24 patients who were included in the RNA-seq analysis. On average, 73% of the TCL1A^+^ cells expressed CD19 (range: 39-95%), and an average of 23% of these TCL1A^+^CD19^+^ cells also expressed CD20 (range: 0-84%).

Taken together, the majority of TCL1A^+^ cells expressed CD19 and CD10; in contrast, a minority of cells expressed CD20, and relatively few cells expressed CD79a or DNTT. Most of the DNTT^+^ cells did not express CD10. Interestingly, the CD19^+^ B cells were distributed throughout the stroma, although some were organized in B cell structures adjacent to tumor epithelial fields.

### Correlation between B cells, TCL1A expression and patient survival

We examined the correlations between patient survival and TCL1A^+^CD19^+^ cells, TCL1A^+^CD19^+^CD20^−^ cells and TCL1A^+^CD19^+^CD20^+^ cells on the FFPE samples (*n* = 24). A high (i.e., above the median of 31 cells/mm^2^) number of TCL1A^+^CD19^+^ cells showed a trend towards improved survival, similar to the correlation for the total number of TCL1A^+^ cells (see Figure 4C). We found no significant correlation between TCL1A^+^CD19^+^CD20^+^ cells or TCL1A^+^CD19^−^CD20^−^ cells and patient survival (data not shown), suggesting that TCL1A^+^CD19^+^ cells play the most clinically relevant role.

**Figure 6:**
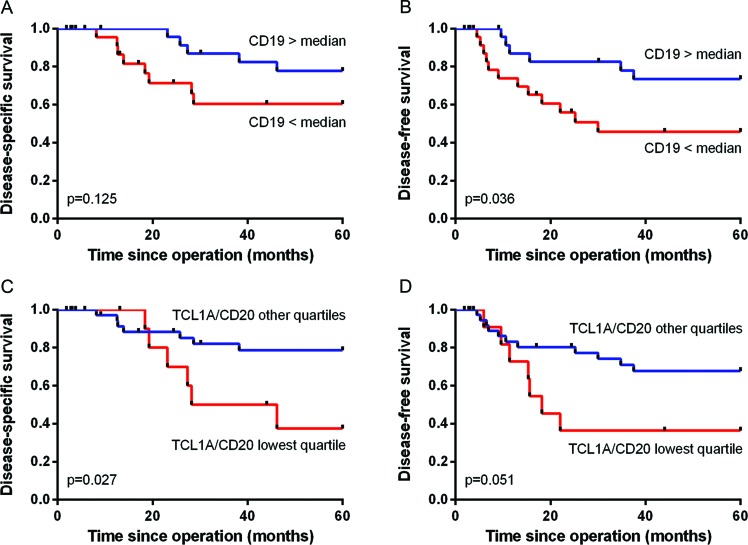
Correlation between B cell and TCL1A expression and survival **A.** and **B.** Kaplan-Meier survival curves and log-rank survival analyses based on qRT-PCR analyses of fresh-frozen tumor samples. The correlation between high versus low CD19 expression and disease-specific **A.** and disease-free survival **B.** is shown. **C.** and **D.** The ratio of *TCL1A*/*CD20* expression was divided in quartiles, and the correlation between the lowest quartile versus the other three quartiles and disease-specific survival **C.** and disease-free survival **D.** is shown.

Next, we used qRT-PCR analysis to validate the correlation between TCL1A and CD19^+^ B cells and improved survival in cervical cancer patients by analyzing the expression levels of *CD19*, *CD20* and *TCL1A* in fresh-frozen cervical cancer samples obtained from 15 patients in the same cohort and an additional 37 samples. We found no significant correlation between *CD19* expression and disease-specific survival (*p* = 0.125; Figure 6A); in contrast, high *CD19* expression was significantly correlated with improved disease-free survival (*p* = 0.036; Figure 6B). High expression of *CD3E*, *CD20* or *TCL1A* was not significantly correlated with survival (data not shown), although a high ratio of *TCL1A*/*CD20* expression showed a trend towards improved disease-specific survival (*p* = 0.053). Based on a quartile division, a low ratio (i.e., the lowest quartile) of *TCL1A*/*CD20* expression was significantly correlated with poor disease-specific survival (*p* = 0.027; Figure 6C) and showed a trend towards poor disease-free survival (*p* = 0.051; Figure 6D). Lastly, the ratio of *TCL1A*/*CD19* expression was not significantly correlated with survival.

## DISCUSSION

Tumors contain several cell types, including tumor cells, fibroblasts, endothelial cells and infiltrating immune cells. This heterogeneous nature of tumors can complicate the expression analysis of cell subpopulations in the tumor. Here, we report the first study in which RNA was isolated separately from viable tumor epithelial cells and tumor-infiltrating immune cells using flow-sorted fresh-dissociated squamous cell cervical carcinomas. Our aim was to use this novel approach in an attempt to identify factors that are correlated with the clinical outcome of cervical cancer patients.

Differential gene expression analysis of the RNA-seq data revealed that the expression profile in the tumor cell fractions differed from the expression profile in the immune cell fractions. In addition, the most differentially expressed genes matched the epithelial cell and leukocyte origins of the flow-sorted fractions, thus validating the study approach. Interestingly, the most significantly differentially expressed genes were upregulated in the immune cell fractions as compared with the tumor cell fractions. One possible explanation for this finding is that the immune cells in these tumors are a highly heterogeneous population of cells characterized by a higher number of function-specific proteins. The tumor epithelial cells on the other hand might represent a more homogeneous cell population characterized by a more uniform set of proteins.

The lack of differentially expressed genes within the tumor cell fractions based on survival status suggests that the tumor samples were highly homogeneous. We could thus not identify a differential expression pattern based on survival status. Because of this relative homogeneity, we believe that the technique used is a promising tool for identifying pathways that are specific to different cancer types.

Our approach identified *TCL1A* as a novel putative biomarker for predicting survival in patients with cervical cancer. Specifically, *TCL1A* was not expressed in the immune cell fractions of the deceased patients. We validated the correlation between high *TCL1A* expression and improved survival using both qRT-PCR and IHC analyses. Despite the relatively small number of patients in our study, *TCL1A* expression was robustly correlated with improved survival in the flow-sorted immune cell fractions. A clear trend towards improved prognosis was also found based on the number of TCL1A^+^ cells in the FFPE samples obtained from the same patients that were included in our RNA-seq analysis. Noh *et al*. reported the presence of TCL1A protein in cervical cancer cells [17], which we did not observe in our patient samples. The reason for this discrepancy might be the use of different antibodies. Since that study did not find a correlation between TCL1A expression and survival, strong lymphocyte-specific expression might be specifically correlated with patient survival.

TCL1A is expressed in immature CD4^−^CD8^−^ T cells and in several stages of developing B cells, including pre-B cells, virgin, mantle and germinal center B cells [18–20]. TCL1A is also expressed in activated peripheral lymphocytes, promoting cell proliferation and survival by activating protein kinase B (Akt) [21]. In humans and mice, overexpression of TCL1A due to chromosomal rearrangement of the *TCL1A* gene to the T cell receptor locus causes mature T cell leukemia and lymphoma [22, 23]. Indeed, TCL1A overexpression is a common finding in both leukemia and lymphoma [23], although amplification of this gene was also reported in a pre-malignant cervical lesion [24]. In addition, Hoyer *et al*. showed that TCL1A expression induces proliferation by increasing T cell receptor signaling in mature T cells [25]. We found that TCL1A was not expressed by T cells in our patient samples. In contrast, TCL1A was expressed predominantly in CD19^+^ and CD10^+^, CD34^−^ B cells. Some of these cells also expressed CD20 and—in rare cases—some cells expressed CD79a or the pro/pre B cell marker DNTT. Based on these markers, we conclude that the TCL1A^+^ B cells seem to be predominantly germinal center or mature B cells, as well as a subpopulation of pre or immature B cells.

Specifically, the presence of TCL1A^+^CD19^+^ cells was correlated with a trend towards improved survival. Remarkably, high *CD19* expression was significantly correlated with improved disease-free survival (based on fresh-frozen tumor tissue samples obtained from an additional cervical cancer patient cohort), whereas *CD3E*, *CD20* and *TCL1A* were not significantly correlated with survival. A low ratio of *TCL1A*/*CD20* expression was significantly correlated with poor disease-specific survival, indicating that high *TCL1A* expression relative to the expression of the B cell marker *CD20* in total tumor samples is correlated with improved survival. These results suggest that CD19^+^ B cells are an important determinant of clinical outcome, and they suggest that the TCL1A^+^ and CD20^+^ B cell populations play an essential role in this outcome. Further validation on an independent patient cohort is required for TCL1A to be used as a prognostic marker.

TCL1A^+^ B cells were distributed throughout the stroma, although they were also organized in B cell structures located adjacent to tumor epithelial fields. We speculate that TCL1A^+^ B cells might indicate lymphoid follicular processes that facilitate B cell maturation, somatic hypermutation and isotype switching. Recently, intratumoral lymphoid structures were reported in several types of cancer [26, 27], and the presence of these structures was correlated with improved survival [28, 29]. Compared to T cells, the role of B cells in cancer has been studied less extensively and is controversial. In addition to producing antibodies, tumor-infiltrating B cells can also function as antigen-presenting cells [30]. In melanoma and breast cancer mouse models, CD19^+^CD20^+^CD137L^+^ B cells were reported to activate cytotoxic T cells [31, 32]. High numbers of tumor-infiltrating B cells have been correlated with improved patient survival in several cancer types [33, 34]. Moreover, antibodies can undergo antigenic selection and affinity maturation in cervical cancer [35]. Our data indicate that intratumoral B cells may play an important role in controlling cervical cancer, and TCL1A is a potential marker for a beneficial B cell response.

In summary, we report that RNA-seq data can be analyzed reliably using tumor cells and immune cells isolated from cancer samples using flow cytometry. The most distinguishing gene identified by differential expression analysis was *TCL1A*. Specifically, *TCL1A* was not expressed in the immune cells of patients who died from cervical cancer. This correlation between *TCL1A* expression and improved survival was validated using qRT-PCR and IHC analyses. We also found that TCL1A was predominantly expressed in B cells, possibly reflecting intratumoral lymphoid follicular processes. Based on these findings, we conclude that TCL1A may be a prognostic biomarker for predicting improved survival in patients with cervical cancer. As B cell expressed *TCL1A* was the most prominent marker correlated with patient survival, perhaps we should broaden our T cells biased view to include B cells in cancer immunology. The emerging interest in cancer immunotherapy supports studying the use of B cells in anticancer treatments.

## MATERIALS AND METHODS

### Patient material

Tumor samples were obtained from patients who underwent primary surgical treatment for squamous cell cervical cancer from 1985 through 2005. Samples with sufficient material to dissociate and freeze cells available in the archives of the Department of Pathology (Leiden University Medical Center, Leiden, the Netherlands) were selected for this study (*n* = 36). None of the patients received preoperative therapy, and clinical and follow-up data were obtained from patient medical records. Samples were handled in accordance with the medical ethical guidelines described in the Code of Conduct for the Proper Secondary Use of Human Tissue established by the Dutch Federation of Biomedical Scientific Societies.

### Tumor tissue dissociation and sample preparation

Fresh tumor samples were dissociated as described previously [36–38]. In brief, the tumor tissue was rinsed in RPMI medium (Gibco Life Technologies, Bleiswijk, the Netherlands) and subsequently dissected into 1-2 mm^3^ pieces, which were incubated overnight in 10 ml DMEM (Gibco) containing 0.5 mg/ml collagenase type II and 0.002% DNase I type II (both from Sigma-Aldrich, St. Louis, MO). The suspension was then incubated in 0.25% trypsin (Gibco) for up to one hour at 37°C until a single-cell suspension was obtained. The cells were then filtered through a 100-μm pore size nylon sieve (Verseidag-Industrietextilien GmbH, Kempen, Germany), and 10% (v/v) fetal calf serum (FCS) was added. The cells were frozen in 90% FCS/10% DMSO at −180°C until further use.

The protocol for preparing the samples for cell sorting was adapted from Tighe and Matthew [39]. Cell suspensions were thawed rapidly in a 37°C water bath, added to a total volume of 10 ml RPMI medium and centrifuged at 1000x*g* at 4°C for 3 minutes. The cell pellet was washed in 100 U RNasin Ribonuclease Inhibitor (Promega, Madison, WI) in 1 ml phosphate-buffered saline (PBS), filtered through a 50-μm filter (BD Biosciences, San Jose, CA), stained with haematoxylin and counted. Depending on the total number of cells in each sample, 1-6 million cells were transferred to RNase-free tubes and centrifuged at 800x*g* at 4°C for 5 minutes. The cell pellets were resuspended in RNasin/PBS, RNasin/PBS containing 1:10 FITC-labelled mouse anti-CD45 antibody (clone T29/33; Dako, Glostrup, Denmark), 1:10 PE-labelled mouse anti-CD326 antibody (i.e., anti-EpCAM; clone EBA-1; BD Biosciences), or RNasin/PBS containing both antibodies. The cells were then incubated in the dark at 4°C for one hour. The samples were washed three times in 1 ml RNasin/PBS by centrifuging at 800x*g* at 4°C for 5 minutes. The pellets were then resuspended in RNasin/PBS containing 1:4000 TO-PRO-3 (Life Technologies, Carlsbad, CA) and transferred to RNase-free flow cytometry compatible tubes. Identical samples were pooled.

### Flow cytometry

A FACSARIA IIu (BD Biosciences) was used for cell sorting. Prior to each sorting run, all parts of the sort chamber were cleaned with RNaseZap (Life Technologies), and the inlet tube and flow cell were cleaned with 10% Contrad70 followed by RNasin/PBS. Dead cells were excluded based on TO-PRO-3 staining [40]. Blue and red lasers were used for excitation. A live gate was set using a FSC-A versus TO-PRO-3 fluorescence dot plot. A cut-off of 3% living TO-PRO-3^−^ cells was used to exclude samples from further analysis (7 samples exceeded this cut-off and were excluded). Cell doublets were excluded using FSC-H versus FSC-W and SSC-H versus SSC-W pulse-processing. Single-stained samples were used to correct for spectral overlap. EpCAM^+^ and CD45^+^ single-positive cells in the double-stained samples were sorted using a 16,16 purity mode, a 100-μm nozzle at 20 psi and a drop frequency of 30.8 kHz. A representative example of the restricted gating strategy for sorting pure EpCAM^+^ tumor epithelial cells and CD45^+^ immune cells is shown in Figure 1. Cells were collected in 2-ml tubes containing 1 ml ice-cold RNAprotect Cell Reagent (Qiagen, Hilden, Germany), mixed and kept on ice until RNA isolation.

### Total RNA sequencing

RNA was isolated and DNA was removed using the RNeasy Plus Micro kit (Qiagen). The RNA was eluted from the column with two rounds of 14 μl RNase-free water. RNA concentration was measured using a Nanodrop device, and RNA integrity was analyzed using RNA 6000 Pico chips in a model 2100 Bioanalyzer (Agilent Technologies, Santa Clara, CA). A cut-off of 100 ng RNA and an RNA integrity number (RIN) of 8 were used to exclude samples with an insufficient amount of RNA and/or insufficient quality RNA (5 samples were excluded based on these criteria). The mean RIN value of the included samples was 9.2 (range: 8.3-10.0). 20 U RNasin was added to each sample, and the samples were stored at −80°C until use.

External RNA Controls Consortium (ERCC) EXfold spike-in mix (Life Technologies) was added for quality control to 50 ng of total RNA. The RNA was then converted to cDNA and pre-amplified using the SMARTer Ultra Low RNA kit (Clontech Laboratories, Mountain View, CA). A sequencing library was generated from 1 ng amplified cDNA using the Illumina Nextera XT kit (Illumina, San Diego, CA) as described previously [41]. Paired end 100-base pair sequencing was performed using a HiSeq 2000 v3 (Illumina). FastQ files were generated from each sample using CASAVA version 1.8.2 (Illumina).

### RNA-seq data analysis

Sequencing adapters in the FastQ files were detected using FastQC version 0.10.1 and removed using the cutadapt tool (version 1.4.2). Base quality trimming was performed using the sickle tool (version 1.200). RNA was aligned to a custom index based on the hg19 human genome (GSNAP version 2014-05-15; novel splicing flag set to true), supplemented with the ERCC spike-in sequences and replacing the published mitochondrial chromosome with the Revised Cambridge Reference Sequence (GenBank acc. no. NC_012920_1). The resulting alignment file was compressed, indexed and name-sorted using the samtools tool (version 0.1.19-44428cd). Reads mapping to ribosomal RNA (obtained from the UCSC rmsk track on the hg19 genome) were removed using a custom-written script. The count table was generated using htseq-count (HTSeq suite version 0.6.1p1; —format flag: BAM; —stranded flag: no; —order flag: name; —mode flag: intersection-nonempty). The UCSC genePredToGtf generated RefSeq annotation (raw database dump of the refGene table) was used as a GTF reference. One of the immune cell fractions was excluded from differential expression analysis because the correlation between the external RNA controls was insufficient (R < 0.9).

Differential expression analysis was performed using the R [42] package edgeR [43]. This package uses an overdispersed Poisson model to account for both biological and technical variability. Empirical Bayes methods were used to moderate the degree of overdispersion across transcripts, enhancing the reliability of inference [43]. The values were normalized using the trimmed mean of M (TMM) and the respective library sizes [44], after which the expression differences between two groups were estimated. Reported p values are false discovery rate—corrected p values calculated using the Benjamini-Hochberg procedure. The Database for Annotation, Visualization, and Integrated Discovery (DAVID; david.abcc.ncifcrf.gov) was used to identify enriched GO terms [45].

### qRT-PCR analysis

RNA was isolated and cDNA was synthesized from four 20-μm slides prepared of 52 fresh-frozen squamous cell cervical cancer samples (obtained from 15 patients who were included in the RNA-seq analysis and an additional 37 samples), described previously [46]. cDNA was also synthesized from 150 ng of the RNA from the remaining immune cell fractions. The resulting cDNA from the immune cell fractions was purified using the QIAquick PCR Purification Kit (Qiagen). Sybr Green-based qRT-PCR was performed in duplicate using 1:25 diluted cDNA from the sorted immune cell fractions or 1:125 diluted cDNA from the fresh-frozen tumor samples and 3 pmol RT² qPCR Primer Assay for amplifying human *CD19*, *EEF1A1*, *MS4A1* (*CD20*), *RPLP0* or *TCL1A* (Qiagen) on a CFX384 system (Bio-Rad, Hercules, CA). Amplification of *ACTB* was performed using the forward 5′-GCCCTGAGGCACTCTTCCA-3′ and reverse 5′-CGGATGTCCACGTCACACTTC-3′ primers designed using Primer-BLAST [47]. As a negative control, the cDNA was replaced with milli-Q water. The most stably expressed genes (*EEF1A1* and *RPLP0*) were used for normalization. Expression was scaled by standard deviation. For statistical analysis, gene expression levels were divided into four quartiles, and the lowest quartile (i.e., low expression) was compared with the other three quartiles; in addition, the lower two quartiles were compared with the upper two quartiles.

### Immunohistochemistry

Immunostaining was performed as described previously [7] on 4-μm thick FFPE sections obtained from the patients included in the RNA-seq analysis. In brief, deparaffinized sections were treated with 0.3% H_2_O_2_ in methanol for 20 minutes to block endogenous peroxidase activity. After rehydration, antigen retrieval was performed in 10 mM Tris and 1 mM EDTA (pH 9.0). The samples were incubated in monoclonal rabbit anti-TCL1A (EPR3949, Abcam, Cambridge, UK) diluted in 1% (w/v) bovine serum albumin (BSA) in PBS at room temperature overnight. The samples were then incubated in BrightVision poly-horseradish peroxidase (HRP) anti-mouse/rabbit/rat antibody (Immunologic, Duiven, the Netherlands) at room temperature for 30 minutes. HRP activity was visualized using 3,3′-diamino-benzidine-tetrahydrochloride (DAB+, Dako). The sections were counterstained with haematoxylin and mounted using CV Mount (Leica Biosystems, Newcastle, UK). The number of strong TCL1A^+^ cells was counted in two to four hot-spots at the center of the tumor using an Axioskop-20 microscope equipped with a Plan-Neofluar 20x/0.5 objective (Zeiss, Göttingen, Germany), sampling a total tumor area of 1.6-3.1 mm^2^ comprising vital tumor epithelium and stroma. For statistical analysis, the number of positive cells was divided into two groups based on the median of 56 cells/mm^2^.

Double- and triple-fluorescent IHC was used to stain TCL1A in combination with mouse IgG1 anti-CD3 (clone F7.2.31, Dako), mouse IgG2b anti-CD8 (clone 4B11, NCL-L-CD8-4B11, Leica Biosystems), mouse IgG1 anti-CD10 (clone 56C6, Dako), mouse IgG1 anti-CD19 (clone 3B10, LifeSpan Biosciences, Seattle, WA), mouse IgG2a anti-CD20 (clone L26, Dako), mouse IgG1 anti-CD34 (clone QBEnd 10, Dako), mouse IgG1 anti-CD79a (clone JCB117, Dako), and/or mouse IgG2a anti-DNTT (clone 41C, Abcam); all incubated at room temperature overnight. The samples were then incubated in a combination of Alexa Fluor-labelled goat anti-rabbit-A488 (A11008), goat anti-mouse IgG1-A647 (A21240), goat anti-mouse IgG2a-A546 (A21133), and/or goat anti-mouse IgG2b-A546 (A21143; all from Life Technologies) at room temperature for one hour. As negative controls, the primary antibodies were replaced with antibodies of the same isotype with unknown specificity. Images were acquired using an LSM700 confocal laser-scanning microscope equipped with an LCI Plan-Neofluar 25x/0.8 Imm Korr DIC M27 objective (Zeiss). Two to four random images were obtained, sampling a total tumor area of 0.5-1.0 mm^2^ comprising vital tumor epithelium and stroma. Double and triple positivity of cells was determined using LSM Image Browser software (version 4.2.0.121, Zeiss). The numbers of TCL1A^+^, CD19^+^, and CD20^+^ single-, double-, and triple-positive cells were scored manually in TCL1A^+^ cell hot-spots at the center of the tumor using ImageJ version 1.47 (http://rsb.info.nih.gov/ij). For statistical analysis, the number of positive cells was divided into two groups based on the median of 43 TCL1A^+^ cells/mm^2^, 31 TCL1A^+^CD19^+^ cells/mm^2^, 18 TCL1A^+^CD19^+^CD20^−^ cells/mm^2^ and 5 TCL1A^+^CD19^+^CD20^+^ cells/mm^2^.

### Statistical analysis

Correlations between qRT-PCR and IHC data and both disease-specific survival and disease-free survival were analyzed using Kaplan-Meier curves in SPSS version 20.0 (IBM, Armonk, NY). Differences in expression between two groups were analyzed using the independent Student's *t*-test. All tests were performed two-sided, and differences with a *p*-value < 0.05 were considered statistically significant.

## SUPPLEMENTARY MATERIAL FIGURE AND TABLES






