# Supplementary material for: Whole-transcriptome analysis of flow-sorted cervical cancer samples reveals that B cell expressed TCL1A is correlated with improved survival
Source: Oncotarget. 2015 Jun 19;6(36):38681–94. doi: 10.18632/oncotarget.4526 (PMC4770729; doi:10.18632/oncotarget.4526)
Supplement: Supplementary file 1 [file oncotarget-06-38681-s001.pdf]

Whole-transcriptome analysis of flow-sorted cervical cancer samples reveals that B cell expressed TCL1A is correlated with improved survival

Supplementary Material

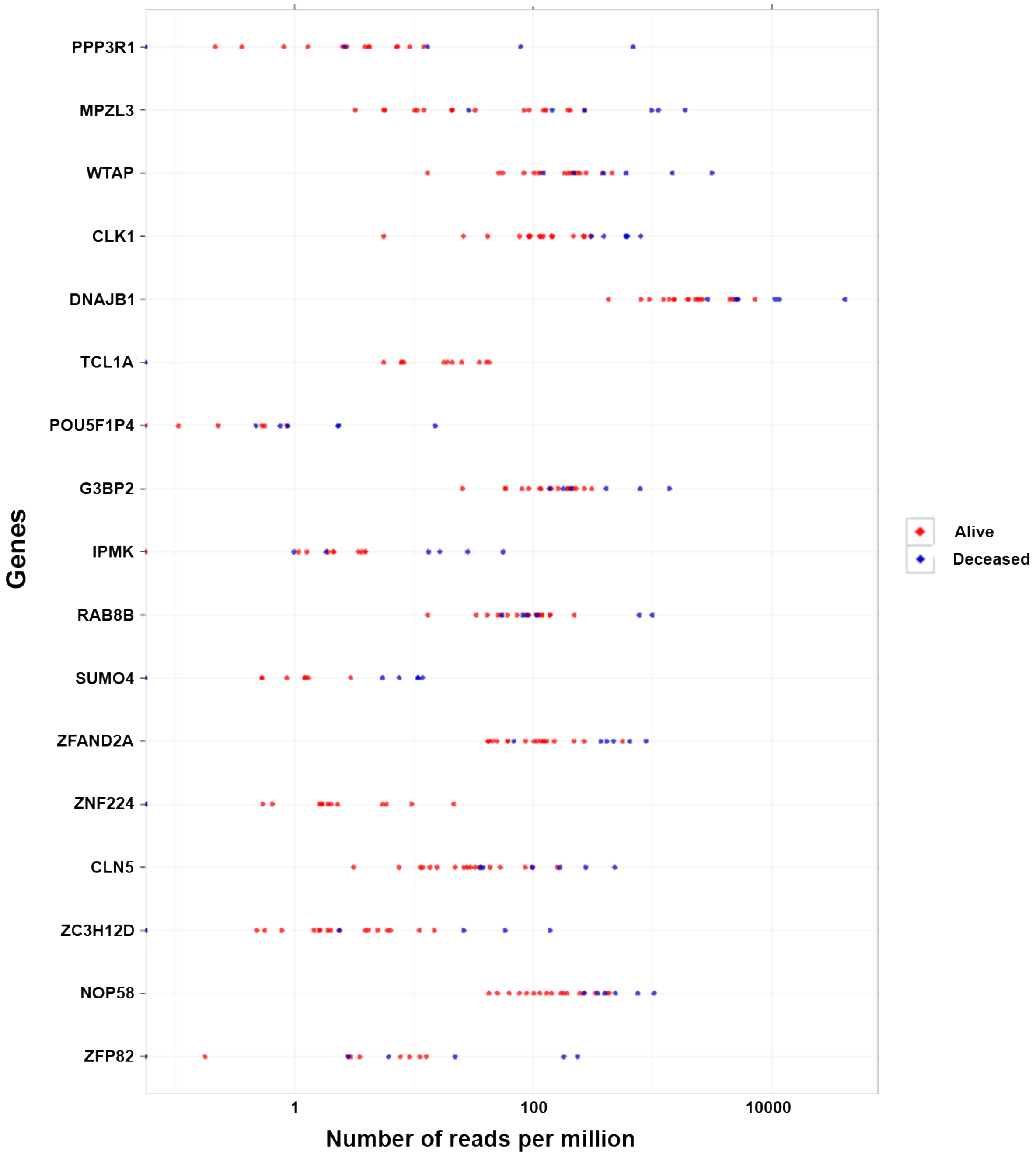

Supplemental Figure S1. Genes differentially expressed within the immune cell fractions based on patient survival status.

The genes significantly differentially expressed in the tumor-infiltrating immune cell fractions between patients who were alive (red dots) and patients who were deceased (blue dots) are shown. The gene with the lowest p value (*PPP3R1*) is shown at the top.
